# Broadband Time-Resolved Absorption and Dispersion Spectroscopy of Methane and Ethane in a Plasma Using a Mid-Infrared Dual-Comb Spectrometer

**DOI:** 10.3390/s20236831

**Published:** 2020-11-29

**Authors:** Muhammad Ali Abbas, Luuk van Dijk, Khalil Eslami Jahromi, Mohammadreza Nematollahi, Frans J. M. Harren, Amir Khodabakhsh

**Affiliations:** Trace Gas Research Group, Department of Molecular and Laser Physics, Institute of Molecules and Materials, Radboud University, 6525 AJ Nijmegen, The Netherlands; Luuk.vandijk@student.ru.nl (L.v.D.); Kh.Eslami@science.ru.nl (K.E.J.); M.Nematollahi@science.ru.nl (M.N.); F.Harren@science.ru.nl (F.J.M.H.); A.Khodabakhsh@science.ru.nl (A.K.)

**Keywords:** dual frequency comb spectroscopy, mid-infrared absorption and dispersion spectroscopy, electrical discharge plasma, time-resolved plasma kinetics

## Abstract

Conventional mechanical Fourier Transform Spectrometers (FTS) can simultaneously measure absorption and dispersion spectra of gas-phase samples. However, they usually need very long measurement times to achieve time-resolved spectra with a good spectral and temporal resolution. Here, we present a mid-infrared dual-comb-based FTS in an asymmetric configuration, providing broadband absorption and dispersion spectra with a spectral resolution of 5 GHz (0.18 nm at a wavelength of 3333 nm), a temporal resolution of 20 μs, a total wavelength coverage over 300 cm^−1^ and a total measurement time of ~70 s. We used the dual-comb spectrometer to monitor the reaction dynamics of methane and ethane in an electrical plasma discharge. We observed ethane/methane formation as a recombination reaction of hydrocarbon radicals in the discharge in various static and dynamic conditions. The results demonstrate a new analytical approach for measuring fast molecular absorption and dispersion changes and monitoring the fast dynamics of chemical reactions over a broad wavelength range, which can be interesting for chemical kinetic research, particularly for the combustion and plasma analysis community.

## 1. Introduction

The conversion of ethane and methane to other hydrocarbons in plasmas has been extensively studied, both theoretically and experimentally. Different non-thermal plasma techniques, such as direct current (DC), MW or RF discharge, dielectric barrier discharge (DBD), and corona or spark discharge, have been demonstrated for this purpose under various conditions [1,2,3,4,5,6,7,8]. The aim of these studies is to develop an efficient process, for converting small hydrocarbons, such as methane, to other valuable hydrocarbons. For this, it is particularly advantageous to find an efficient non-thermal (non-equilibrium) plasma operation, since it requires less energy and can operate at lower temperatures as compared to thermal (equilibrium) plasmas [9]. To characterize the plasma process and maximize the conversion efficiency, various molecular species and reaction processes have to be measured and monitored during plasma operation. For this, methods are employed based on mass spectrometry [10] and optical detection, for the latter, specifically emission/absorption spectroscopy [11,12,13,14,15]. The main advantage of optical-based detection is their fast response time, in-situ measurement, and species specificity. Among the many laser-based gas spectroscopic methods, plasma diagnosis in the mid-infrared wavelength region (2–20 µm) is of great interest, since strong fundamental, ro-vibrational transitions of most molecules are present in this fingerprint wavelength region. 

The coherent mid-infrared sources for probing these fundamental transitions can be either narrowband or broadband. In the past, narrowband, tunable lead-salt diode lasers have been applied for plasma diagnosis, detecting mid-infrared active molecular species like hydrocarbons, fluorocarbon, boron, etc. A comprehensive review can be found in [16] and references therein. The main disadvantage of using lead-salt lasers is their need for cryogenic cooling and narrowband tuning range (~7 cm^−1^), limiting the number of detectable species. Recent advances in the continuous-wave (CW) and pulsed quantum cascade lasers (QCL) and interband cascade lasers (ICL) led to the greater flexibility and compactness of mid-infrared sources for plasma diagnostics. These narrowband sources are tuned over single molecular absorption lines, with high spectral resolution and sensitivity owing to their continuous tunability and high spectral brightness. External cavity quantum cascade lasers (EC-QCL) have made it possible to probe the molecular transitions of multiple species in a wide spectral range (~300 cm^−1^). However, plasma processes include complex chemical reactions at different timescales, involving molecular species such as neutrals, ions, and radicals, each with their distinct spectral feature. In order to monitor these reactions, a high temporal resolution and a broad spectral bandwidth are required simultaneously; this is not possible with cw sources. Time-resolved broadband spectra can be recorded using pulsed broadband sources in combination with a Fourier transform spectrometer (FTS) [17] or a dispersive-based spectrometer [18,19]. The use of such sources for plasma diagnostics is comprehensively discussed elsewhere [20,21]. 

Over the past decade, dual-comb spectroscopy (DCS) [22,23,24] has demonstrated the possibility of simultaneously providing a broad spectral bandwidth with high spectral resolution in a short measurement time. Subsequently, mid-infrared DCS [25,26,27,28,29] has emerged as an alternative technique to traditional FTS. In DCS, the interferogram is made by the interference of femtosecond pulses from two mode-locked lasers, each generating a frequency comb, which have a slightly different repetition frequency with respect to each other. Similar to traditional FTS, the interferogram is recorded using a single-point detector and the absorption spectrum is achieved by a Fourier Transformation of the recorded interferogram. Due to the lack of mature, mode-locked lasers in the mid-infrared wavelength range, non-linear conversion systems like different frequency generation (DFG) or optical parametric oscillation (OPO) are used to generate mid-infrared frequency combs for DCS [27,30,31,32,33]. Recently, mode-locked QCLs have also been used for mid-infrared DCS, and demonstrated promising spectroscopic results [26,34]. As such, mid-infrared DCS have received increasing attention for various applications, such as combustion diagnostics [35,36,37], study of protein dynamics [38], radical-radical interactions in flash photolysis mixtures [39], and DC/pulsed plasma discharges [40].

In order to measure dispersion spectra, in addition to absorption spectra, dispersive FTS and DCS in an asymmetric arrangement have been demonstrated in the past [31,34,41,42,43,44,45,46]. For this, the sample is placed in one arm of the interferometer, and an asymmetric interferogram is recorded with a single detector; hence the name asymmetric/dispersive FTS. This technique can provide molecular absorption and dispersion spectra from the complex refractive index of the sample. In our previous work, we have developed a mid-infrared DCS system and demonstrated its performance using a discharge plasma [40]. Here, we have modified our DCS in an asymmetric arrangement to measure time-resolved absorption and dispersion spectra, simultaneously. We use this to study methane and ethane in a DC discharge, under static and dynamic conditions. The spectra are recorded with a spectral resolution of 5 GHz (0.18 nm at a wavelength of 3333 nm), a spectral bandwidth of 300 cm^−1^ (2850–3150 cm^−1^), and a time resolution of 20 µs. To the best of our knowledge, this is the first demonstration of broadband, time-resolved, absorption/dispersion spectroscopy in the mid-infrared wavelength range for plasma diagnostics. This approach opens up new opportunities in plasma diagnostics. Simultaneous monitoring the phase and absorbance of species in plasma processes are particularly useful for quantifying their complex susceptibility and refractive index, indicating their plasma densities [47]. 

## 2. Materials and Methods

### 2.1. Absorption and Dispersion Spectroscopy

A monochromatic electromagnetic (light) wave probing a gas sample experiences a change in phase and amplitude, at a frequency that is in resonance with a molecular transition. This is given as:

(1) $$E=E_{0}e^{i\left(\omega t-k_{0}n_{a}l\right)},$$

where $E_{0}$ is the amplitude of the electric field, ω the angular frequency of the light, $k_{0}$ the wave vector pointing in the propagation direction, l the interaction length of light with the sample, and $n_{a}$ the complex refractive index of the sample near a molecular transition. E is generally a function of the frequency of light, v=ω/2π. At a particular time (t=0):

(2) $$E=E_{0}e^{i\left(-k_{0}n_{a}l\right)},$$

Near the molecular resonance, the complex refractive index is given as [48]:

(3) $$n_{a}\left(v\right)=n+n^{\prime }\left(v\right)-i\kappa \left(v\right),$$

in which n is the non-resonant refractive index and $n^{\prime }\left(v\right)-i\kappa \left(v\right)$ is the complex resonant refractive index, in which $n^{\prime }\left(v\right)$ represents the dispersion and κ(v) is the extinction coefficient, the latter quantifies the absorption of the light wave, appearing in Lambert-beer law as $I=I_{0}e^{-\left(2k_{0}\kappa \left(v\right)l\right)}$.

The phase change, ∆φ (in radians), experienced by the light in the medium, is proportional to the dispersion $n^{\prime }\left(v\right)$ and is given by:

(4) $$∆\varphi =2k_{0}n^{\prime }\left(v\right)l,$$

and the absorbance of light is given by:

(5) $$\alpha l=2k_{0}\kappa \left(v\right)l$$

Dispersion spectroscopy has two main advantages:

(i) The intensity of dispersion lines is always proportional to the amount of gas species, whereas absorption lines can saturate at strong absorptions and high concentrations. Therefore, phase spectroscopy provides a larger dynamic range to measure highly absorbing samples.
(ii) Dispersion spectra can be immune to intensity fluctuations, as the phase depends only on the refractive index and not on the laser intensity [49].

For recording molecular absorption and dispersion simultaneously, spectroscopic methods are based on tunable CW lasers [48] using frequency modulation spectroscopy (FMS) [50], noise-immune cavity-enhanced optical heterodyne molecular spectroscopy (NICE-OHMS) [51,52], heterodyne phase sensitive dispersion spectroscopy (HSPDS) [53], chirped laser dispersion spectroscopy (CLaDS), [54] or Faraday rotation spectroscopy (FRS) [55]. Fourier transform spectroscopy (FTS) [56] and Vernier spectroscopy [57] are two broadband methods capable of extracting absorbance and phase changes of samples, simultaneously. Two common FTS methods for this purpose are dispersive Fourier transform spectroscopy (DFTS) [41] and dual comb spectroscopy (DCS) [31,44]. In DFTS, the sample is placed in one arm of the Michelson interferometer and a single-sided, asymmetric interferogram is obtained on the detector, in the time domain by scanning the retro-reflecting mirror of the FT spectrometer. Both absorption and dispersion can be obtained after applying a fast Fourier transform (FFT). Similarly, in DCS this information can be obtained by placing the sample in one of the frequency comb beams, while the other comb beam bypasses the sample, before being combined with the first comb on the detector. As mentioned earlier, the main advantage of DCS over a mechanical FTS is the lack of moving mirrors, which provides very fast data acquisition with high spectral and temporal resolution.

### 2.2. Experimental Setup

**Figure 1:**
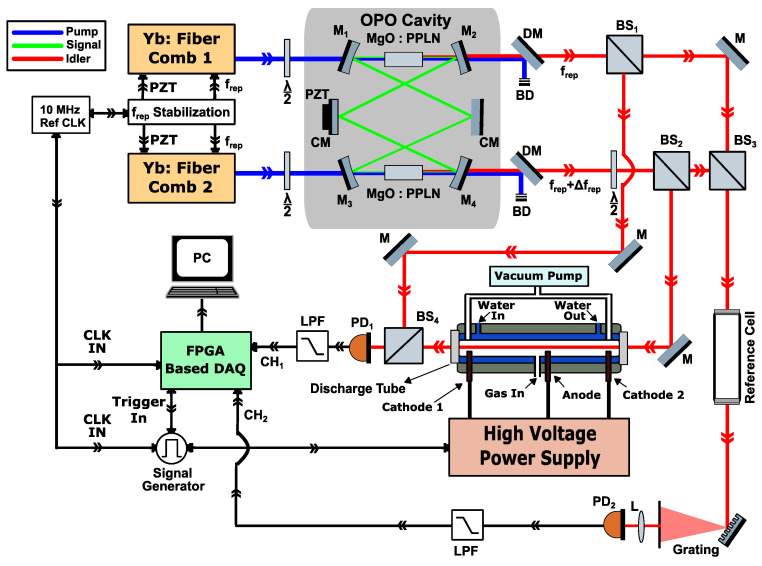
Experimental setup for mid-infrared dual-comb absorption and dispersion spectroscopy in an electrical discharge plasma. M_1–4_, curved cylindrical mirrors; CM, chirped mirrors; PZT, piezoelectric transducer; BS_1_ and BS_2_, pellicle beam splitter for beam division; BS_3_ and BS_4_, pellicle beam splitters for beam combination; BD, beam dumps; DM, dichroic mirrors; PD_1,2_, HgCdTe photodetectors.

Figure 1 demonstrates the schematic of the experimental setup. It consists of the mid-infrared dual-comb source and the plasma discharge system. Here, we explain each sub-system, as well as the data acquisition and signal processing in the following subsections. 

#### 2.2.1. Mid-Infrared Dual-Comb Source

Two near-infrared, Yb-fiber-based, femtosecond mode-locked lasers (1064 nm, Menlo Systems, Planegg, Germany), with stabilized repetition frequency (*f*_rep_, ~90 MHz) and free-running carrier-envelope offset frequencies (*f*_ceo_), are used as pump sources for two singly-resonant, optical parametric oscillators (OPOs) sharing a single ring cavity [31]. For Dual Comb Spectroscopy (DCS), one pump comb has a slightly different repetition frequency (*f*_rep_) with respect to the second pump comb, so that the difference in the repetition frequency (Δ*f*_rep_) can be set between 200 and 300 Hz. A 10 MHz reference clock is used for referencing all of the frequency sources (for *f*_rep_ control), triggering the data acquisition, and modulating the discharge [40,58].

The two mode-locked lasers are orthogonally polarized, using half-wave plates before entering the OPO in a counter-propagating manner. Two 5-mm-long periodically poled Lithium Niobate (PPLN) crystals (Covesion Ltd., Romsey, UK), containing eight poling periods, are used in the OPO. The OPO has a ring cavity configuration and consists of four concave mirrors (AR coated at 1064, and 3650–4850 nm, HR coated at 1350–1500 nm, Layertec GmbH, Mellingen, Germany) with a focal length of 5 cm, and six flat, chirped mirrors (HR coated 1370–1750 nm, Layertec GmbH), the latter six for dispersion compensation (for simplicity Figure 1 shows two chirped mirrors). One of the chirped mirrors is mounted on a translational stage, connected to a piezo-electronic transducer (PZT) for coarse and fine-tuning of the length of the OPO cavity. The cavity length was adjusted around 330 cm to match the repetition frequencies (*f*_rep_) of both near-infrared pump lasers (~90 MHz), enabling both of the counter-propagating signal beams to be in resonance with the cavity modes [31]. Two orthogonally polarized mid-infrared beams are generated from the OPO along with collinear residual pump beams. Two dichroic mirrors (HR coated at 1064, and HR coated at 3200–3900 nm, Layertec GmbH) are used to separate idler and pump beams. The two idler beams have a spectral bandwidth (full width half maximum) of ~300 cm^−1^ and a maximum of 200 mW average power. A half-wave plate is used for rotating the polarization of one mid-infrared beam to align both polarizations.

#### 2.2.2. Dual-Comb Spectrometer Design for Absorption/Dispersion Spectroscopy

**Figure A1:**
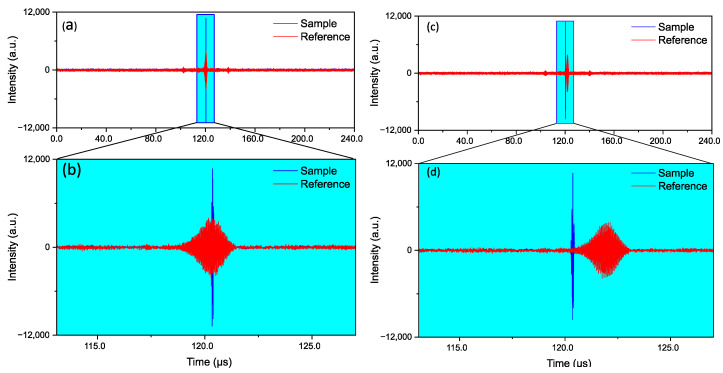
(**a**) Measured sample and reference interferograms with perfectly balanced beam paths. (**b**) Zoom into the central parts of the interferograms given in (**a**). (**c**) The influence of 3 mm CaF_2_ window in the path of one frequency comb beam before the beam splitter, causing a shift in the sample interferogram in time domain with respect to the reference interferogram. (**d**) The time shift can be seen by zooming into the central part of the interferograms.

Each idler beam is split into two channels, called the sample and reference channels. A 50-cm-long discharge tube is placed in the sample channel, whereas in the reference channel, a 30-cm-long absorption cell is placed for optical frequency calibration. For the splitting of each beam, we use two 50:50 beam splitters (BS_1_ and BS_2_) and two 50:50 beam splitters (BS_3_ and BS_4_) for recombination of the beams. In the sample channel, the idler beam of comb-2 passes through the discharge tube, while the idler beam of comb-1 bypasses the tube, and the two beams are combined on BS_4_. In the reference channel, the two idler beams are combined by BS_3_, and afterward both pass through the reference gas cell (containing pure methane at 100 mbar). As such, the sample channel has an asymmetric configuration (providing both absorption and dispersion spectra), while the reference channel is symmetric (only yielding the absorption spectra). The asymmetric interferogram of the sample channel is detected by an HgCdTe photodetector (PD_1_, 50 MHz bandwidth, PVI-4TE Vigo System SA, Ożarów Mazowiecki, Poland), as shown in Figure A1 in the Appendix A (in blue). The reference absorption cell is used for absolute frequency calibration of the sample spectrum. The output beam from the reference cell is dispersed with the help of a diffraction grating, and a mechanical slit is used to band-pass-filter the spectrum around a single line of methane centered at 3038.5 cm^−1^ before focusing the beam on a HgCdTe photodetector (PD_2_, 50 MHz bandwidth, PVI-4TE Vigo System SA). We use the optical frequency position of this reference absorption line for removing the f_ceo_ fluctuations and frequency calibration of the dual-comb spectrum. More details on absolute frequency calibration can be found in [58].

The femtosecond pulses need to arrive at the same time on the detectors, therefore the lengths of sample and reference channels are adjusted in such a way that both idler combs travel an equidistant path, from the generation point in the non-linear crystal to the recombination beam splitters, i.e., BS_3_ and BS_4_. This way, we avoided any relative time-delay between sample and reference interferograms, and record both in the same time-frame of the data acquisition. Figure A1c,d in the Appendix A show the effect of an induced displacement in the time domain between the sample and reference interferograms inserting a 3 mm thick CaF_2_ window in the path of one of the beams. It clearly shows the sensitivity of the optical layout to path length difference. Therefore, well-matched optical path lengths are necessary; however, no active control of the path lengths is required. 

#### 2.2.3. Discharge System and Gas Mixtures

As shown in Figure 1, the experimental setup for creating the discharge, in static DC and dynamic operation, consists of a discharge tube, a High Voltage power supply (Haefely Hipotronics, Brewster, NY, USA), and an external signal generator (33500B, Agilent, Santa Clara, CA, USA) for modulating the current of the HV power supply. The glass discharge tube is 50 cm long with a 3 mm internal diameter. Water (at 23 °C) is flowing around the discharge tube at a constant flow rate to control the temperature of the discharge tube during operation. The discharge is split at the anode (in the center) towards two hollow cathodes at both ends of the tube. The anode is connected to the high voltage (HV) power supply (DC voltage 25 kV, current 40 mA). Each cathode is connected in series with a current regulator (containing ballast resistors) for limiting the current within acceptable bounds through the discharge tube. 

For the discharge experiments, we used a gas mixture of methane in helium (40:60 ratio) and mixture of ethane in helium (33:67 ratio), and studied the dynamics in absorption and dispersion spectra of the reactants, and produced species, as a result of the discharge. For the methane-helium mixture experiment, a constant flow rate of 1.5 Nl/h (normal liters/hour) was applied using flow controller, while for ethane-helium mixture a flow rate of 3 Nl/h was used. In both cases, a total pressure of 25–30 mbar was maintained in the discharge, using a pressure controller and a vacuum pump after the discharge tube. The inlet of the gas mixtures was chosen to be near the anode, whereas the outlets are placed near the cathodes at both ends, as shown in Figure 1. A static discharge operation was obtained by keeping discharge current at a constant level. For dynamic discharge operation, the discharge current was modulated by a square wave (on-off). A dual-channel signal generator (33500B, Agilent), referenced to the same 10 MHz reference clock that is used for stabilizing the repetition rate of the two frequency combs, is employed to generate the square-wave modulation (at 265 Hz, 6.6% duty cycle). The second output of the signal generator is connected to the external trigger input of the data acquisition card to provide synchronization of the data acquisition and discharge pulses, as described in the next section. 

#### 2.2.4. Data Acquisition and Synchronization 

A LabVIEW-based program controls the data acquisition from the spectrometer. The hardware for fast data acquisition consists of a two-channel AD convertor (NI-5762, National Instruments, Austin, TX, USA) combined with a Field Programmable Gate Array module (FPGA, PXIe-7962R National Instruments). The interferograms from the sample and reference photodetectors are low pass filtered (at 50 MHz) and sampled with a rate of 125 MSample/s, recording 30,000 data points on each channel, yielding a single-shot acquisition time of $T_{acq}=$ 240 µs. The recorded interferograms are stored in first-in-first-out (FIFO) buffers in the FPGA and transferred to the host PC immediately, after recording each sample and reference interferogram. Further data processing is performed offline using a MATLAB-based program. For microsecond time-resolved measurements, the dual-channel signal generator (synchronized to the reference clock) is used to modulate the discharge by its first output channel and provides a trigger signal for the DAQ card on its second output channel. The second output channel of the signal generator can be manually delayed compared to the first one, providing a tunable time-delay between the discharge modulation and the triggering of the data acquisition. Therefore, transient discharge dynamics can be probed at different, relative time-delays, with respect to the discharge modulation. We used a minimum time-delay step of 20 µs to record the interferograms. The time-delay is fixed for each time-step (data acquisition time ~3 s, used for averaging the spectra), such that the entire period of the discharge modulation was probed at different time delay intervals, by a step-scanned mechanism. A more detailed description of the timing scheme and synchronization can be found in [40]. 

#### 2.2.5. Data Processing

The recorded interferograms are time-stamped and post-processed using a Matlab-based program. A Blackman apodization function is applied to the interferograms in the post-processing to improve the signal-to-noise ratio and remove ringing effects around the narrow absorption lines in the sample and reference spectra [58]. The Blackman apodization function *A*(*x*) is given by:

(6) $$A\left(x\right)=\frac{21}{50}+\frac{1}{2}cos\left(\frac{\pi x}{a}\right)+\frac{2}{25}cos\left(\frac{2\pi x}{a}\right),$$

in which *x* denotes the *x*-coordinates of the interferogram (the individual sampling points) and *a* denotes the total number of samples in the interferogram before apodization i.e., 30,000.

After apodization, a fast Fourier transform (FFT) is applied to each reference and sample interferogram yielding the reference and sample spectra in the RF domain. The spectra are then converted to the optical domain via the corresponding scaling factor ($f_{\mathrm{rep}}$/$∆f_{\mathrm{rep}})$. A shot-to-shot variable frequency shift remains in the measured spectra, due to the $f_{\mathrm{ceo}}$ fluctuations on both mid-infrared combs. To remove these shifts, we use the known position of an absorption line in the reference spectrum (~3038.5 cm^−1^) and correct for the shifts in both dispersion and absorption spectra of the sample. The absorption and dispersion spectra are averaged after the shift correction, yielding high SNR, and absolute-frequency-calibrated spectra [58]. 

## 3. Results and Discussion

### 3.1. Proof of Principle

**Figure 2:**
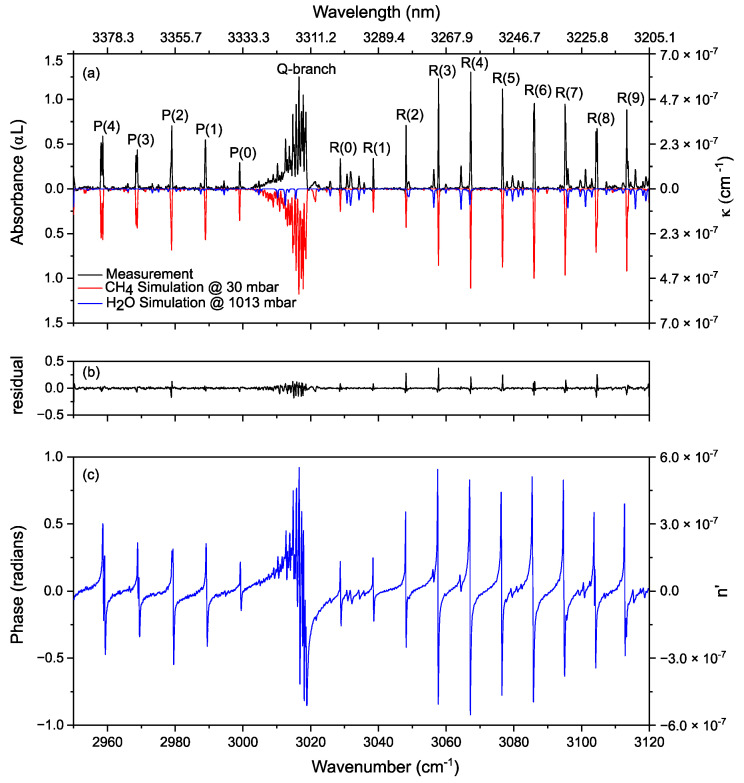
(**a**) Absorbance spectrum of ν_3_ band of methane diluted in helium (10% CH_4_) at 30 mbar and atmospheric water (in black), along with a theoretical model spectrum of CH_4_ ν_3_ band (in red, inverted for clarity) and water (in blue, inverted for clarity) using HITRAN database parameters. Calculated extinction coefficient (right ordinate axis) from the measured absorbance using Equation (5) (**b**) The residual showing the satisfactory fit of simulation to the measurement. (**c**) Measured phase spectrum of 10% CH_4_ diluted in He (at 30 mbar) and water (at 1 atmosphere) along with calculated dispersion (right ordinate axis) from the measured data of phase using Equation (4).

We evaluated the performance of the experimental setup by measuring the absorption and dispersion spectrum of the ν_3_ band of methane diluted in helium (10:90 ratio) at 30 mbar. Figure 2a shows the measured absorbance spectrum (in black, an average of 700 shots, 240 µs acquisition time for each single shot), as well as the modeled methane (in red, inverted for clarity) and water absorbance spectrum (in blue, inverted for clarity). The water spectrum is modeled for an interaction length of 275 cm at atmospheric pressure; i.e., the distance from the output of the OPO cavity till the photodetector (PD_1_). The modeled spectra are calculated from the HITRAN 2016 database [59], using a Voigt profile and convolving a Blackman instrument line-shape function corresponding to the applied apodization function to the interferogram (Equation (6)). To achieve the concentrations, we fit the modeled spectra to the measured spectrum and remove the baseline and etalon fringes from the measurement, by adding a low order polynomial and few low-frequency sine waves to the fit [60]. A methane concentration of 10.8% and a water concentration of 1.05% were retrieved from the fit, with a spectral resolution of 5 GHz and a spectral precision of 120 MHz [58]. Figure 2b shows the observed-fit residual with a 2.72% standard deviation at the noise level. Although the measured and modeled spectra are generally in good agreement with each other, there are some remaining features in the residuals. This is due to the narrow absorption lines of methane at low pressure, where the linewidths are comparable to the calibration precision of the system.

Figure 2a also shows the extinction coefficient κ(v) on the right ordinate axis, calculated from the absorbance data using Equation (5). Figure 2c shows the measured phase (dispersion) spectrum of the gas sample and atmospheric water retrieved from the same data set as the absorbance spectrum. The resonance dispersion features, corresponding to the absorption peaks of methane and water, are easily recognizable. The recorded phase is a direct measure of the molecular dispersion $n^{\prime }\left(v\right)$, which is shown on the right ordinate axis in Figure 2c. 

To calculate the noise equivalent absorption sensitivity (NEAS) of the spectrometer, we took the ratio of two consecutive spectra, filling a sample cell (*L* = 50 cm) with helium, and fit and remove the baseline and etalon fringes as in the case of absorption spectra. The noise in the wavelength range of 2950–3120 cm^−1^ is *σ* = 5.64 × 10^−2^. Considering the spectral resolution (~0.17 cm^−1^) and the number of spectrally resolved elements (M ≈ 1000), the NEAS = (*σ*/*L*) × (*T*/*M*)^1/2^ = 3.19 × 10^−6^ cm^−1^ Hz^−1/2^ per spectral element. To determine the long-term stability of the system, we averaged the spectra over 3 s, in the white noise dominated regime. This averaging time is well below ~10 s averaging time-limit dictated by the Allen-Werle plot for our spectrometer [58]. 

The measured absorbance shown in Figure 2a can also be used to calculate the absorption cross-section ($\sigma_{v}$ in m^2^/molecule) of methane in a gas sample using:

(7) $$\mathrm{Absorbance}=\alpha l=\sigma_{v}nl,$$

in which n is the particle density of the absorbent gas in molecules/m^3^, given as:

(8) $$n=\frac{P}{k_{B}T}$$

where *P* is the pressure, $k_{B}$ is the Boltzmann constant and, *T* is the thermodynamic temperature.

Hence, the absorption cross-section is:

(9) $$\sigma_{v}\left({\mathrm{cm}}^{2}/\mathrm{molecule}\right)=\frac{\left(10^{4}\alpha k_{B}T\right)}{P}$$

**Figure 3:**
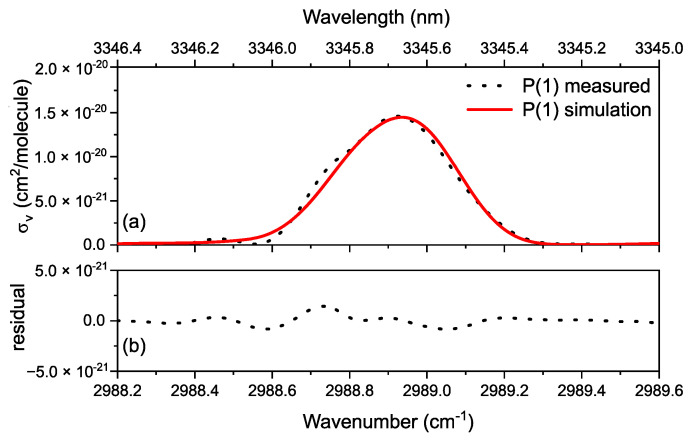
(**a**) Measured (in dotted black) and simulated (in solid red, HITRAN database) absorption cross-section of P(1) line of methane ν_3_ band, calculated for a pressure of 30 mbar (3000 Pascal) and temperature of 296 K. (**b**) The residual shows the fit of measurement to the simulation. The oscillations in the residue are due to the non-negligible error of the frequency calibration compared to the absorption linewidths in unapodized form.

Figure 3a shows the measured and modeled absorption cross-section $\sigma_{v}$ of the P(1) transition in the ν_3_ vibrational band of methane. As the absorption cross-section data is derived from the absorbance data in Figure 2a, it automatically considers the broadening processes in terms of Voigt profile, convolved with Blackman instrumental line shape function. Figure 3b shows the residual of the fit; the small features in the residual are due to the comparable precision of the frequency calibration and the unapodized absorption linewidth. 

### 3.2. Methane in a Static (DC) Discharge 

**Figure 4:**
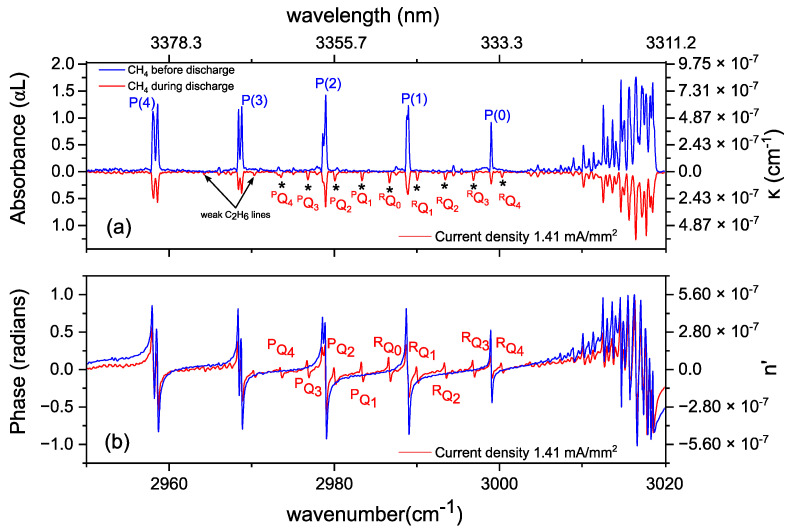
(**a**) Absorption spectra (left ordinate axis) and extinction coefficient (right ordinate axis) of 40% CH_4_ diluted in He, at 25 mbar before (in blue, 600 averages) and during (in red, 600 averages, inverted for clarity) the discharge. Ethane ^R^Q_K_ absorption features of ν_7_ band appearing during the discharge are indicated by “*”. (**b**) Corresponding phase spectra (left ordinate axis) and dispersion n’ (right ordinate axis) of the absorption spectra shown in (**a**).

In this section, we study the absorption and dispersion spectrum of methane in helium (40:60 ratio (25 mbar) in a static discharge at a constant gas flow of 1.5 Nl/h. Before applying the discharge the methane absorption spectrum was measured first (Figure 4a, in blue, 600 averages). Thereafter, we applied a discharge with a stabilized current of 10 mA (DC voltage 10 kV) corresponding to an average current density of 1.41 mA/mm^2^. In the corresponding absorption spectrum (Figure 4a, in red, 600 averages, inverted for clarity), it can be clearly seen that the methane absorption lines decrease in intensity. This is an indication of a reduced ground state population in methane and/or a reduction in methane concentration, due to collisional excitation of the molecule to higher ro-vibrational levels, as well as fragmentation or ionization into neutral radicals and molecular ions. Simultaneously, additional absorption lines appear, indicated by “*” in the red spectrum. These lines correspond to the ^R^Q_K_ branches of the ν_7_ band in ethane (C_2_H_6_), along with weaker lines corresponding to the P and R branch of the ν_7_- and ν_10_-band in ethane [61,62,63]. 

Ethane is produced in the discharge and results from recombination reactions of generated radicals. It should be noted that there is a cascade of chemical reactions in the electrical discharge, generating numerous charged (ions) and neutral species (atoms, radicals, excited molecules). Here, we restrict our discussion to excitation and de-excitation of the observed molecules, their dissociation reaction by electron impact, and their formation via recombination of radicals. The main pathway for the formation of C_2_H_6_ is the dissociation of CH_4_ by an electron impact, followed by recombination of two CH_3_ radicals, given as:

(10) $$e+{\mathrm{CH}}_{4}\to [\cdot {\mathrm{CH}}_{3}]+\left[\cdot H\right]+e\;\;\;\;\;(\mathrm{dissociation})$$

(11) $$[\cdot {\mathrm{CH}}_{3}]+[\cdot {\mathrm{CH}}_{3}]+M\to C_{2}H_{6}+M,\;\;\;(\mathrm{recombination})$$

in which M represents the third collisional partner, due to the conservation of momentum, which can be the surface of the discharge tube or other molecular species.

A more thorough analysis of the chemical reactions requires a detailed study of different dissociation and recombination reactions, rate constants and conversion efficiencies of the species involved in the reaction, which is outside the scope of this work. It can be found elsewhere with various discharge conditions [64,65].

The discharge processes can also be studied via the refractive index of the gas medium. Figure 4b shows the observed phase spectra before (in blue, 600 averages) and during the discharge (in red, 600 averages). Similar to the absorbance spectra, a decrease in the phase is observed at the CH_4_ lines, next to the increase of phase at the C_2_H_6_ ro-vibrational transitions during the discharge. The calculated extinction coefficient κ(v) from the absorbance data before and during the discharge is shown on right ordinate axis of Figure 4a. Similarly, the molecular dispersion is demonstrated on the right ordinate of Figure 4b.

To observe dissociation of methane and formation of ethane with different DC current densities, we increased the current density from 0 to 3.54 mA/mm^2^. Note that a flow rate of 1.5 Nl/h was maintained during the discharge. We introduced a waiting time of one minute before recording various spectra at different current values to avoid transient states.

**Figure 5:**
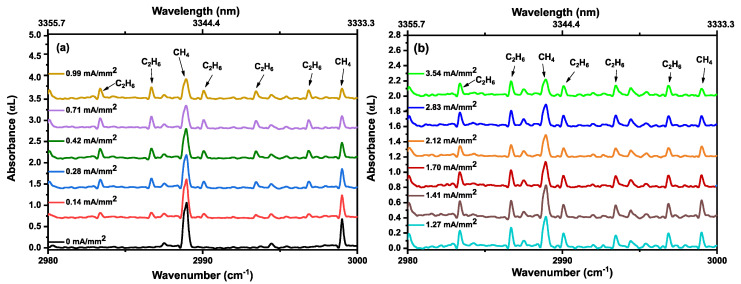
Absorption spectra of 40% CH_4_ diluted in He, at 25 mbar at (**a**) 0 mA/mm^2^ to 0.99 mA/mm^2^ and (**b**) 1.27 mA/mm^2^ to 3.54 mA/mm^2^ discharge current densities, demonstrating the kinetics of methane and ethane absorption features. Consecutive spectra are vertically shifted for clarity.

**Figure 6:**
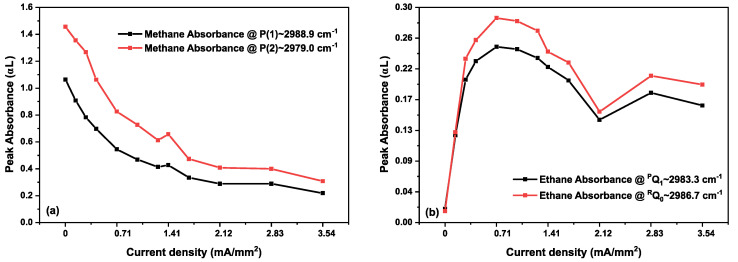
The peaks of two absorption features are plotted for each molecule to show the consistency of the kinetics. (**a**) Kinetics of methane peak absorbance in a 40% CH_4_ diluted in He gas sample at 25 mbar upon increasing discharge current density. (**b**) Kinetics of ethane peak absorbance in the same sample. Solid lines are used to connect the data points for better visibility.

**Figure 7:**
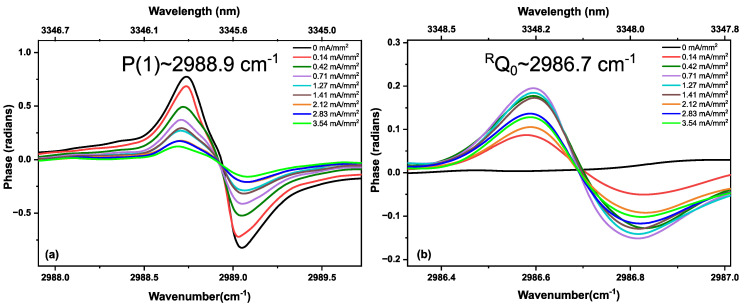
Dispersion (phase) spectra of (**a**) a methane absorption line of the gas sample (40% CH_4_ diluted in He at 25 mbar) and (**b**) an ethane absorption line (produced by the discharge) upon increasing the discharge current density.

As we are only observing methane and ethane kinetics in the discharge, we show the measured absorbance spectra for only a small wavenumber range (from 2980 to 3000 cm^−1^) in Figure 5. Figure 5a,b show the measured absorbance spectra for different discharge current densities, demonstrating CH_4_ and C_2_H_6_ absorption features. A vertical offset shifts the consecutive absorbance spectra for a good visual presentation. Up to a current density of 0.99 mA/mm^2^, a clear decrease in intensity of the CH_4_ absorption lines can be observed. Simultaneously, C_2_H_6_ absorption features appear. From 1.27 to 3.54 mA/mm^2^, a continuous decrease in CH_4_ absorption features is observed, clearly indicating a depopulation of the vibrational ground state. However, C_2_H_6_ formation demonstrates a reduction at higher current densities, before settling onto a semi-stable value. This decrease in C_2_H_6_ indicates that more ethane molecules are exited and/or dissociated than produced. These kinetics are shown in Figure 6, where the peak intensities of two different methane (P(1)~2988.9 cm^−1^and P(2)~2979.0 cm^−1^) and ethane (^R^Q_0_~2986.7 cm^−1^ and ^P^Q_1_~2983.3 cm^−1^) absorption features are plotted as a function of current density, to provide a more clear representation of the kinetics of both molecules. The same kinetics can be found in the phase spectra. Figure 7 shows the phase spectra for the single rotational P(1) line of methane (2988.9 cm^−1^, panel (a), and an ^R^Q_0_ line of ethane (2986.7 cm^−1^, panel (b)) as a function of the current density. The continuous decrease in the phase spectral features of methane (P(1)) from 0 to 3.54 mA/mm^2^ has a similar behavior as for the absorption observed in Figure 6a. Similarly, the phase kinetics of ethane ^R^Q_0_ feature (Figure 7b) shows a similar trend as Figure 6b.

#### Rotational Temperature of Methane in a Static Discharge Plasma

Broadband absorption spectroscopy can also be used to measure the rotational temperature of the species within the discharge. To demonstrate this, we measured the absorption spectrum of methane/helium gas (1:1) mixture at 25 mbar, before and during discharge.

**Figure 8:**
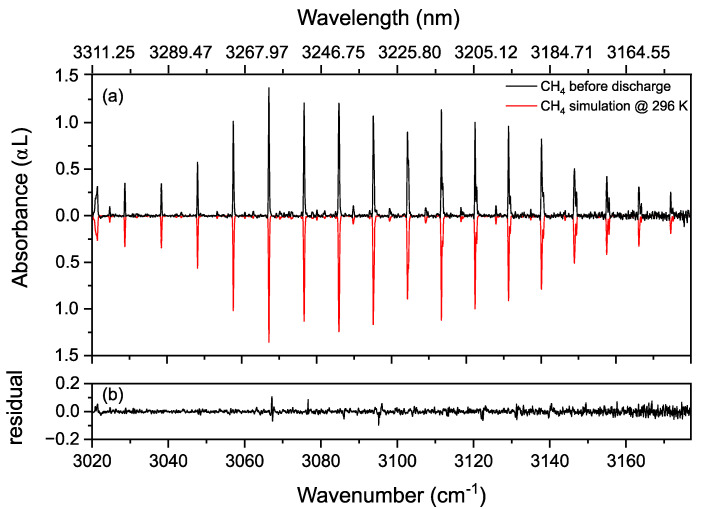

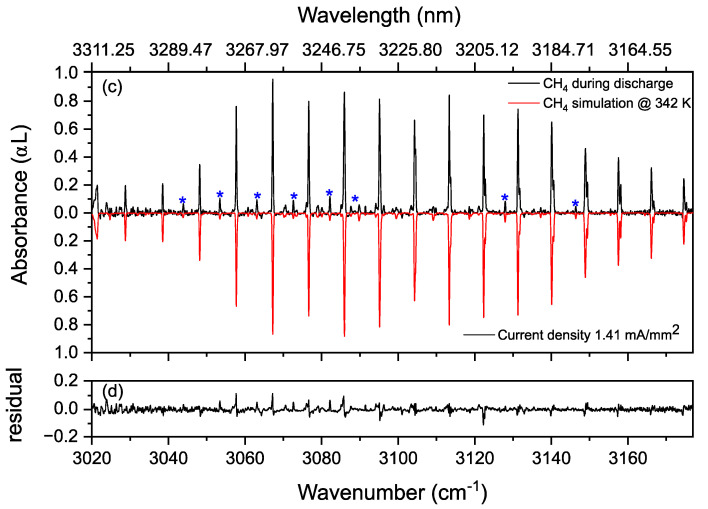
(**a**) Absorbance spectrum of R-branch of ν_3_ band of methane diluted in helium (50% CH_4_) at 25 mbar (in black), along with a fit theoretical model spectrum of 49.7% CH_4_ (in red, inverted for clarity) at 296 K, calculated using HITRAN database parameters. (**b**) The residual of the fit. (**c**) Absorbance spectrum of R-branch of ν_3_ band of methane diluted in helium at 25 mbar during the discharge (in black) along with a theoretical model spectrum of 18.5% CH_4_ (in red, inverted for clarity) at 342 K, calculated using HITRAN database parameters. The Hot bands, appearing during the discharge, are indicated by blue “*”. (**d**) The residual of the fit.

Figure 8a shows the R-branch of the methane ν_3_ band before discharge. The measured (in black) rotational distribution of the R-branch shows a good agreement with the simulated spectrum (in red), calculated using the HITRAN 2016 database with a reference temperature of *T*_ref_ = 296 K [59]. A methane concentration of 49.7% was retrieved from the fitting routine discussed earlier. Figure 8b shows the residual of the fit, indicating a good agreement between the simulation and the measurement.

In the discharge, the methane gas is in a non-thermal equilibrium, resulting in different temperatures for molecular rotations and vibrations. The rotational temperature can be calculated from the measured absorption spectrum (Figure 8c, in black) of the R-branch of methane ν_3_ band (current density of 1.41 mA/mm^2^). To verify the change in rotational distribution we used the simulation model incorporating the total internal partition function, Q, the lower-state energy of the transition, E’’, and the coefficient of temperature dependence of the air-broadening half width, n_air_, of methane from the HITRAN 2016 database [59,66]. Figure 8c compares the modeled methane spectrum (in red) and the measured methane spectrum (in black) during the discharge, achieving a rotational temperature of 342 K within a margin of ±4 K. The retrieved concentration of methane in the vibrational ground state from the fit is 18.5%. Figure 8d shows the residual of the fit. The uncertainty of the temperature measurement comes from the combined uncertainties originating from the concentration, pressure, and fitting algorithm. 

Figure 8c also shows the hot band lines of methane (ν_4_- and ν_2_-band, indicated by blue “*”) during the discharge. To populate these bands, we estimate a vibrational temperature of 1800–1900 K.

### 3.3. Ethane in a Static Discharge

**Figure 9:**
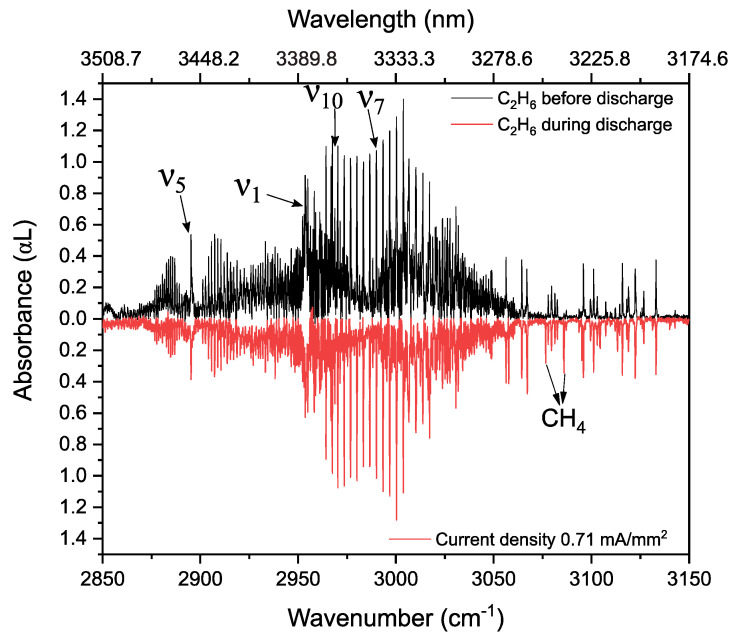
Absorption spectra of 33% C_2_H_6_ diluted in He, at 25 mbar before (in black, 700 averages) and during (in red, 700 averages, inverted for clarity) the discharge.

In this section, we study the absorption and dispersion spectra of ethane in helium (33:67 ratio, flow rate 3 Nl/h, 25 mbar) before and during a static discharge with a stabilized current density of 0.71 mA/mm^2^ (DC voltage~10 kV). The absorbance spectrum before discharge is shown in Figure 9 (in black, 700 averages), and during the discharge in red (700 averages, inverted for clarity). Similar as for methane, two different processes can be observed. The intensity of the ethane lines reduce, indicating vibrational excitation/dissociation of ethane. Next to this, newly formed absorption lines can be observed, although the ethane spectrum is very dense. Some of these are clearly seen between 3050–3150 cm^−1^, and correspond to methane absorption lines; these were identified using the HITRAN database [59]. Two of these features are indicated with arrows in Figure 9.

Methane production in an ethane mixture discharge can be explained as a three-stage reaction. Firstly, the cleavage of the C-C bond of C_2_H_6_ by an electron impact into two CH_3_ radicals (Equation (12)). Secondly, the cleavage of the C-H bond of C_2_H_6_ by an electron impact into C_2_H_5_ and H radicals (Equation (13)), and finally recombination of CH_3_ and H radicals to form CH_4_ (Equation (14)). Note that, this is only one of the pathways of reaction to obtain methane from an ethane discharge, as described in [67],

(12) $$e+C_{2}H_{6}\to [\cdot {\mathrm{CH}}_{3}]+[\cdot {\mathrm{CH}}_{3}]+e\;\;\;(\mathrm{dissociation})$$

(13) $$e+C_{2}H_{6}\to [\cdot C_{2}H_{5}]+\left[\cdot H\right]+e\;\;\;\;\;\left(\mathrm{dissociation}\right)$$

(14) $$[\cdot {\mathrm{CH}}_{3}]+\left[\cdot H\right]+M\to {\mathrm{CH}}_{4}+M\;\;\;\;(\mathrm{recombination})$$

**Figure 10:**
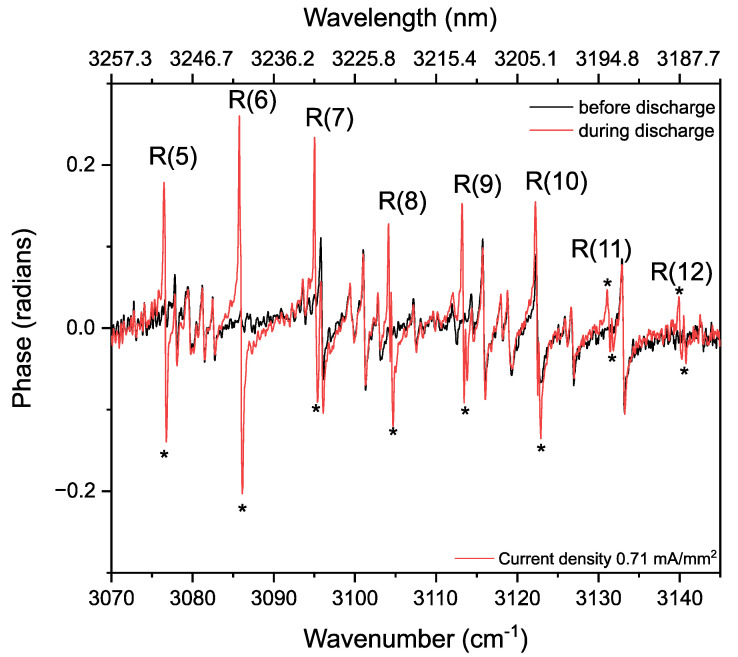
Part of the dispersion (phase) spectrum recorded before (in black, 700 averages) and during the discharge (in red, 700 averages) of 33% C_2_H_6_ diluted in He, at 25 mbar. Methane dispersion features appearing during the discharge are indicated by “*”.

This methane production can also be observed in the phase spectra (Figure 10) between 3070–3145 cm^−1^. The phase features, corresponding to methane transitions, are indicated by “*”. The high SNR phase spectrum helps to distinguish weak features from the noise, which are usually harder to distinguish in the absorption spectrum. 

**Figure 11:**
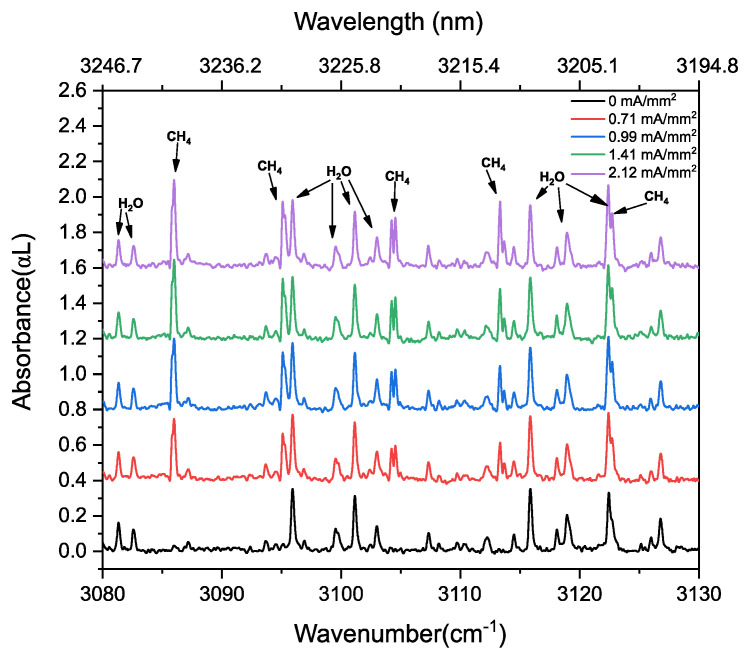
Absorption spectra of 33% C_2_H_6_ diluted in He at 25 mbar at different discharge current densities, demonstrating methane generation. For clarity, the spectra for different current densities are represented at different vertical offsets. Absorption lines of water lines are present due to the transmission of the beams in the atmosphere before and after the discharge cell.

To observe dissociation of ethane and formation of methane at different power levels, we measured the absorbance and phase changes by increasing the current density from 0 to 2.12 mA/mm^2^. Figure 11 shows the absorption spectra at different discharge current densities, demonstrating the ethane dissociation and the subsequent recombination of radicals forming methane as given by three-staged reaction in Equations (12)–(14). Different spectra are given a vertical offset for clarity.

**Figure 12:**
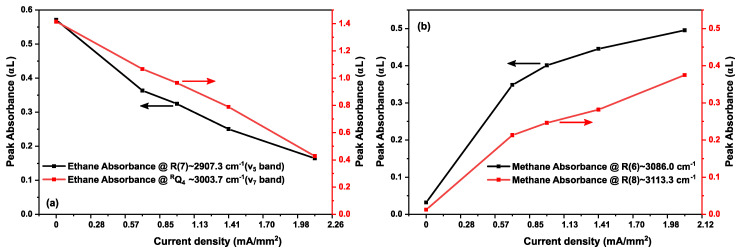
(**a**) Kinetics of ethane peak absorbance in a 33% C_2_H_6_ diluted in He gas sample at 25 mbar upon increasing discharge current density. (**b**) Kinetics of methane peak absorbance in the same sample. Multiple peaks of both molecules are plotted to show the consistency over the vibrational band(s). Solid lines are used to connect the data points for better visibility, and arrows indicate the corresponding ordinate values for different peaks.

**Figure 13:**
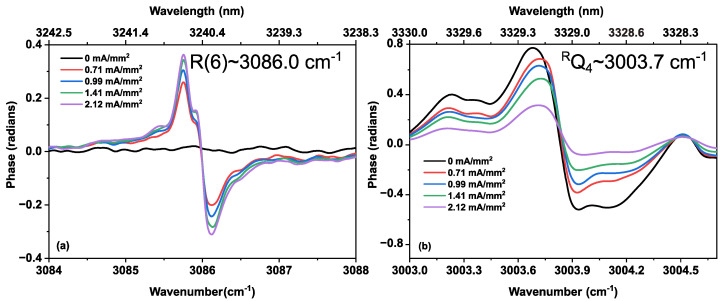
(**a**) Dispersion (phase) spectra of an R(6) line of methane ν_3_ band, produced during the discharge of a gas sample (33% C_2_H_6_ diluted in He, at 25 mbar) and (**b**) dispersion of an ^R^Q_4_ line of ethane ν_7_ band, upon increasing the discharge current density.

To show the kinetics of ethane and methane more clearly, Figure 12 demonstrates the intensities of two methane lines (R(6)~3086.0 cm^−1^ and R(8)~3113.3 cm^−1^ of the ν_3_ band) and ethane absorption lines (R(7)~2907.3 cm^−1^ of ν_5_ band, and ^R^Q_4_~3003.4 cm^−1^ of ν_7_ band) as a function of the discharge current density. Up to a value of 2.12 mA/mm^2^, the absorbance of R(7) of ν_5_ band, and ^R^Q_4_ of ν_7_ band of ethane show a continuous intensity decrease, indicating a depopulation of the vibrational ground states. At the same time, methane starts to form, indicated by the intensity increase of the R(6) and R(8) lines in the ν_3_ band. These trends are also observed in the phase spectra, as shown in Figure 13a,b, for methane and ethane, respectively. As the phase is proportional to the concentration, this increase and decrease of phase spectra indicate the increase and decrease in the concentration of respective molecules during discharge. 

It should be noted that the kinetics of ethane/helium gas mixture were restricted to five current densities from 0 to 2.12 mA/mm^2^. However, this kinetics can be studied for higher current densities as well. As the dissociation and formation of molecules in an electrical discharge depend on parameters such as: applied discharge power, flow rate, and gas pressure, we have only shown the influence of applied power, by varying the current densities. For higher current densities, we predict that the produced methane will settle to semi-stable equilibrium for a constant flow rate and pressure. For current densities beyond 4 mA/mm^2^, we expect further dehydrogenation of methane, as observed in other studies with non-thermal plasma processes [68,69,70]. The dehydrogenation of methane is given as CH_4_→C_2_H_6_→C_2_H_4_→C_2_H_2_. 

### 3.4. Time-Resolved Dynamics at Microseconds Timescale

The rapid acquisition time in dual-comb spectroscopy enables us to monitor fast dynamical processes while changing the discharge current at microsecond time scale. Here, we study the absorption and phase transient dynamics of methane and ethane during a pulsed discharge in a gas mixture CH_4_ in helium (40:60 ratio, 25 mbar, flow rate 1.5 Nl/h). A square wave modulation at 265 Hz and 6.6% duty cycle (on-off ratio) was applied to the discharge current (0.99 mA/mm^2^, 10 KV). As mentioned earlier, the signal generator frequency was equal to, and synchronized (phase-locked) with the difference repetition rate of the two comb sources (Δ*f*_rep_). Since the data acquisition should be synchronized as well, we used the synchronized second channel of the signal generator to trigger the DAQ card. This second channel was manually delayed with respect to the first channel. Therefore, time-resolved measurements could be performed at different triggering times in a step-scan manner. At each time step (step size 20 µs), the interferograms were acquired for 3.3 s (~900 averages), with a single-shot acquisition time of 240 µs per interferogram. The interferograms were measured close to the on/off switching events of the discharge. We increased the step size further away from the switching events, where the discharge processes settle to semi-equilibrium values [40].

**Figure 14:**
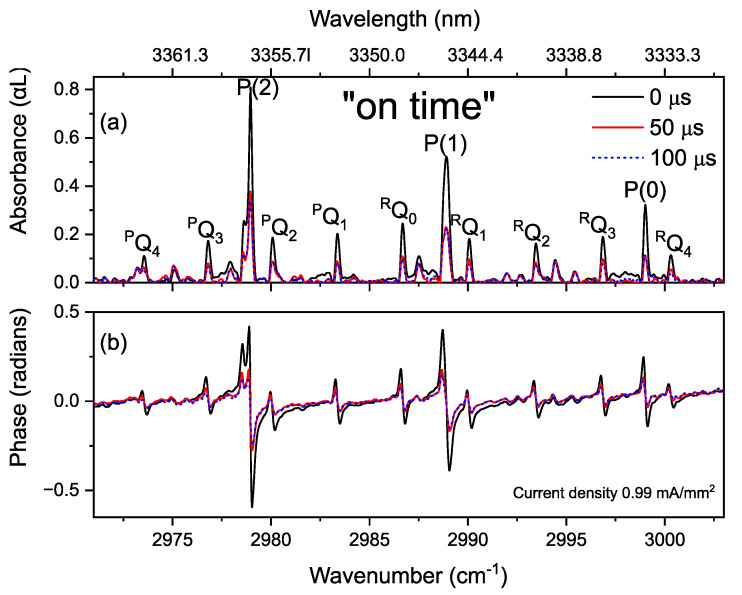

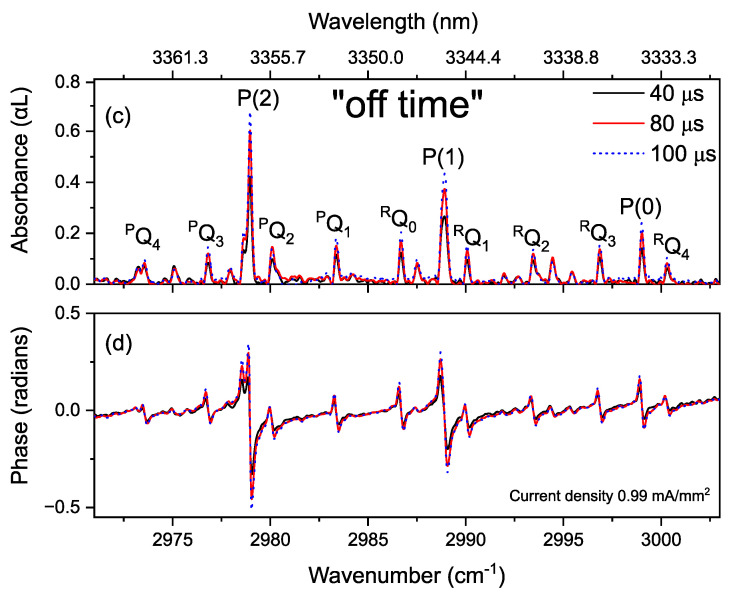
(**a**,**b**) Time-resolved absorption/dispersion spectra of CH_4_ (P(2), P(1) and P(0) lines) and C_2_H_6_ (other lines) at 0, 50, and 100 µs after switching the discharge on. They show a decrease in the concentration of both gas species. (**c**,**d**) Time-resolved absorption/dispersion spectra at the same wavenumber span measured in 40, 80, 100 µs after switching the discharge off, showing an increase in the concentrations, indicating the formation of CH_4_ and C_2_H_6_, via recombination processes of hydrocarbon radicals.

Figure 14 shows broadband, time-resolved absorption and dispersion spectra of methane and ethane, at specific times after switching the discharge on and off. Note that at the start of the discharge, ethane is present in the mixture due to the generation in the previous discharge cycle and is not removed due to the low refresh rate of the gas mixture in the discharge cell. Line by line analysis shows that by turning the discharge on, methane and ethane lines rapidly decrease (Figure 14a,b). The neutral gas species get excited and dissociated. During the off-time of the discharge, the produced radicals recombine forming C_2_H_6_ and CH_4_, as demonstrated by the increase in absorption and dispersion signals (Figure 14c,d). 

**Figure 15:**
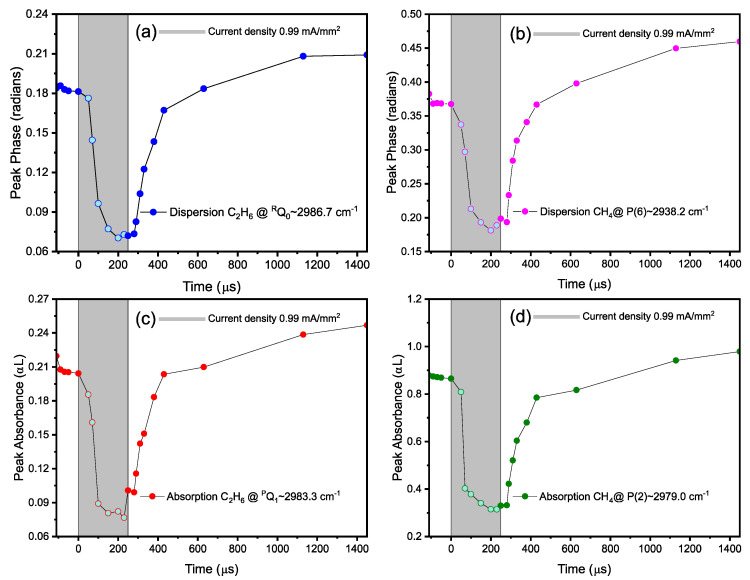
Time-resolved monitoring of the dispersion/absorption features of C_2_H_6_ in (**a**,**c**), and CH_4_ in (**b**,**d**) at microseconds timescale. The discharge “on” time is shown with gray rectangles.

Figure 15 shows the time-profile of dissociation and formation of methane and ethane during a time span of 1560 µs of the total modulation period of ~3773.6 µs, with 250 µs on-time as indicated by a grey box. For the dynamics of both molecules, absorbance and dispersion values are shown for two different methane lines (P(2) and P(6)) and ethane branches (^R^Q_0_ and ^P^Q_1_). Each data point for respective ro-vibrational transition is averaged for ~3.3 s. Therefore, the cumulative measurement time for the entire data set (containing 21 data points) is ~70 s, excluding the standby time for varying the time delay. During the discharge on-time, as shown by a gray box in Figure 15, both methane and ethane show a rapid decrease (~100 µs) due to their excitation, and dissociation into radicals. After the discharge has been switched off, the radicals recombine again, forming methane and ethane. However, in contrast to the dissociation just after switching the discharge on, the recombination process shows a slower exponential rise (~900 µs) in the intensities. 

## 4. Conclusions

It is difficult to obtain broadband time-resolved dispersion spectra, using classical FTS. Here, we demonstrate for the first time, the capability of time-resolved mid-infrared dual comb spectroscopy (DCS), by recording absorption and dispersion spectra from a discharge plasma simultaneously. This recording enables to quantify the complex refractive index of the gas mixture directly over a broad wavelength range. More precisely, we measured the methane decomposition into ethane and ethane decomposition into methane, in terms of absorption and dispersion spectra at various current density of the plasma. In addition, the dynamic behavior of the absorption and dispersion spectra is recorded using a pulsed discharge. The dual comb spectroscopic system covers a spectral bandwidth of 2850–3150 cm^−1^, with a spectra resolution of ~5 GHz, a temporal resolution of 20 µs and NEAS of 3.19 × 10^−6^ cm^−1^ Hz^−1/2^ per spectral element.

Using other poling periods of the PPLN crystal in the OPO, the wavelength range can be extended from 2200 to 4000 cm^−1^, enabling the study of a wide range of molecules, ions, and radicals, which have fundamental ro-vibrational transitions in these wavelength regions. For instance, here we used helium as buffer gas for methane and ethane. Using buffer gases such as nitrogen (N_2_) in combination with methane, will lead to the formation of other compounds such as HCN and NH_3_. To this end, future studies are planned to perform broadband, time-resolved spectroscopy for other species in longer discharges and at higher pressures, which can provide higher sensitivity to detect lower concentrations of molecules and radicals.

**Figure A2:**
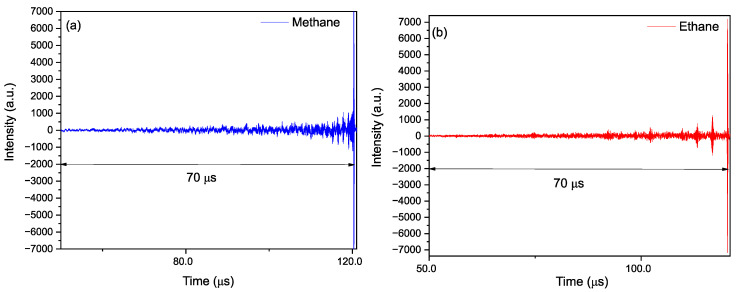
Truncated sample interferograms from methane in (**a**) and ethane (**b**) representing their optical free induction decay (FID).

DCS can also be used to measure the free induction decay of molecular species. By using an asymmetric (one-sided) interferogram, the central burst is in the middle of the interferogram, while the free induction decay (FID) will be present in the tail of the interferogram [71]. A single-shot truncated interferogram of 70 µs showing this phenomenon, is demonstrated in Appendix A Figure A2 for both methane and ethane samples. However, our spectrometer is not capable of time-domain coherent averaging of the interferograms due to the fee running *f*_ceo_ of the combs, therefore the results are limited to single-shot and low SNR FIDs. It would be interesting to use computational coherent averaging [72,73] and/or digital correction [74] methods, to extend the coherence time of the spectrometer and realize a phase-stable dual-comb spectrometer. This will enable the time-domain averaging of the FID signal of the sample and new ways of dual-comb spectroscopy for plasma diagnostics. 

The DCS can also be used for studying fast chemical reactions in the harsh conditions of combustion processes. For example, a fuel/tracer reaction in a continuously scavenged calibration flow cell at high temperature and pressure [75] and to study the dynamics of pyrolysis in combustion systems [76].

The free-running $f_{ceo}$ of the mid-infrared frequency combs constitutes the main limitation of our DCS. To compensate for the fluctuations in the $f_{ceo}$, we used a well-known absorption line of a molecular gas in a reference cell, to calibrate/correct the frequency scale. This procedure determines the stringent requirement of perfectly balanced beam paths, next to a post-processing algorithm to align the spectra before averaging. Therefore, this configuration is more practical for short path lengths in light-matter interaction. Increasing the interaction length using a multipass cell or a resonant optical cavity around the discharge, is possible in this configuration. However, to achieve balanced beam paths a second reference multipass cell or resonant optical cavity is required.
